# Toward the Optimization of (+)-[^11^C]PHNO Synthesis: Time Reduction and Process Validation

**DOI:** 10.1155/2019/4292596

**Published:** 2019-09-30

**Authors:** Sarah Pfaff, Cécile Philippe, Lukas Nics, Neydher Berroterán-Infante, Katharina Pallitsch, Christina Rami-Mark, Ana Weidenauer, Ulrich Sauerzopf, Matthäus Willeit, Markus Mitterhauser, Marcus Hacker, Wolfgang Wadsak, Verena Pichler

**Affiliations:** ^1^Department of Biomedical Imaging and Image-guided Therapy, Division of Nuclear Medicine, Medical University of Vienna, Vienna, Austria; ^2^Institute of Organic Chemistry, University of Vienna, Vienna, Austria; ^3^Department of Psychiatry and Psychotherapy, Division of General Psychiatry, Medical University of Vienna, Vienna, Austria; ^4^Ludwig-Boltzmann-Institute Applied Diagnostics, Vienna, Austria; ^5^CBmed GmbH-Center for Biomarker Research in Medicine, Graz, Austria

## Abstract

(+)-[^11^C]PHNO, a dopamine D_2/3_ receptor agonistic radiotracer, is applied for investigating the dopaminergic system via positron emission tomography (PET). An improved understanding of neuropsychiatric disorders associated with dysfunctions in the dopamine system and the underlying mechanism is a necessity in order to promote the development of new potential therapeutic drugs. In contrast to other broadly applied ^11^C-radiopharmaceuticals, the production of this radiotracer requires a challenging four-step radiosynthesis involving harsh reaction conditions and reactants as well as an inert atmosphere. Consequently, the production is prone to errors and troubleshooting after failed radiosyntheses remains time consuming. Hence, we aimed to optimize the radiosynthesis of (+)-[^11^C]PHNO for achieving better activity yields without loss of product quality. Therefore, we synthesized (+)-[^11^C]PHNO and omitted all heating and cooling steps leading to higher activity yields. As a result, radiosynthesis fully conducted at room temperature led to a time-reduced production procedure that saves about 5 min, which is an appreciable decay-prevention of around 15% of the activity yield. Additionally, we established a troubleshooting protocol by investigating reaction intermediates, byproducts, and impurities. Indeed, partial runs enabled the assignment of byproducts to their associated error source. Finally, we were able to generate a decision tree facilitating error detection in (+)-[^11^C]PHNO radiosynthesis.

## 1. Introduction

**Figure 1:**
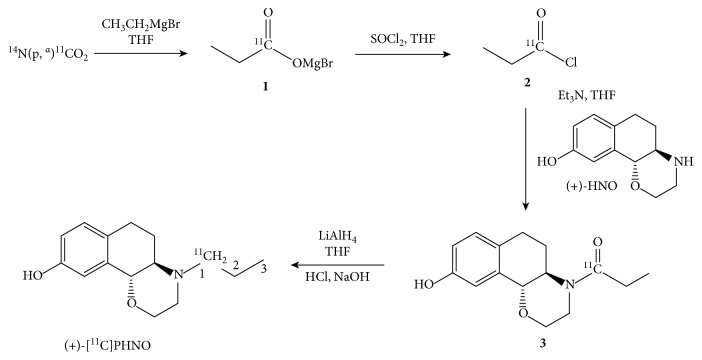
Synthesis scheme for the four-step radiosynthesis of (+)-[^11^C]PHNO starting from cyclotron-produced [^11^C]CO_2_ and ethylmagnesium bromide.

The dopamine system is a key player in many neuropsychiatric disorders like schizophrenia, Parkinson's disease, or attention-deficit hyperactivity disorder [1–3]. (+)-PHNO ((+)-4-propyl-3,4,4a,5,6,10b-hexahydro-2H-naphtho[1,2-b][1,4]oxazin-9-ol hydrochloride) is a well-described agonist for the dopamine D_2/3_ receptor subtypes [4–6]. This compound binds to dopamine D_2_ receptors in their high affinity state and has high affinity for the dopamine D_3_ receptor subtype, making it a virtual D_3_ selective probe in the ventrobasal striatum and brainstem dopaminergic nuclei [4]. It is one of the most important imaging agents measuring changes of extracellular dopamine, as it can be easily displaced by endogenous dopamine in so-called competition experiments, where dopamine-releasing agents such as amphetamine are administered [6, 7]. (+)-[^11^C]PHNO shows high potential to bring new insights into poorly understood physiological processes of a variety of neuropsychiatric disorders. Recently, the application of this tracer in healthy subjects and patients suffering from schizophrenia showed an enhanced dopamine release in patients with schizophrenia [8, 9]. The synthesis of ^11^C-radiopharmaceuticals is highly challenging due to the relatively short half-life of carbon-11 (20.4 min). Consequently, a prerequisite for achieving a reasonable activity yield is a reliable production within two to three half-lives [10]. Furthermore, a fundamental requirement for this particular radiotracer is high molar activity as pharmacological doses cause side effects, like nausea [11]. (+)-[^11^C]PHNO radiosynthesis has been published by several working groups describing synthetic routes via carbon-11 radiolabeling either on the 1-position or the 3-position of the propyl moiety (Figure 1) [12–14]. To the best of our knowledge, [3-^11^C]-(+)-PHNO radiosynthesis has not become widespread, whereas [1-^11^C]-(+)-PHNO is widely applied in clinical studies [15–17]. Radiolabeling of the common (+)-[1-^11^C]PHNO (further stated as (+)-[^11^C]PHNO to be in accordance with the literature) starts with generation of [^11^C]propionic acid chloride, which can be obtained by a Grignard reaction from [^11^C]CO_2_ and ethylmagnesium bromide and subsequent conversion with thionyl chloride (SOCl_2_) (Figure 1, compounds **1** and **2**) [18–20]. This highly reactive acyl chloride readily reacts with the precursor ((+)-HNO) to give an intermediate amide (Figure 1, compound **3**). Finally, the amide is reduced and the product is purified by semipreparative high-performance liquid chromatography (HPLC). However, this radiolabeling procedure is very demanding as inert conditions and extremely harsh reagents are required. Therefore, the automated synthesis needs high technical effort. As an alternative to the conventional vessel-based approach, our working group reported an in-loop method for (+)-[^11^C]PHNO synthesis. This method facilitates the establishment of (+)-[^11^C]PHNO in other PET facilities [21].

Although this method improved the production of (+)-[^11^C]PHNO, the susceptibility to humidity as well as oxygen contamination remains an unsolved problem. As a consequence, (+)-[^11^C]PHNO synthesis has the highest failure rate within our facility. After a failed production run, an extensive validation process starts for investigating which of the numerous steps led to the radiosynthetic failure. During troubleshooting, the impact of the reaction temperature needs special considerations. Wilson et al. reported that the reaction temperature regulated the formation of byproducts: the reduction of the carboxyl-group at temperatures below −30°C results in a lower number of side products compared to higher temperatures [12].

**Figure 2:**
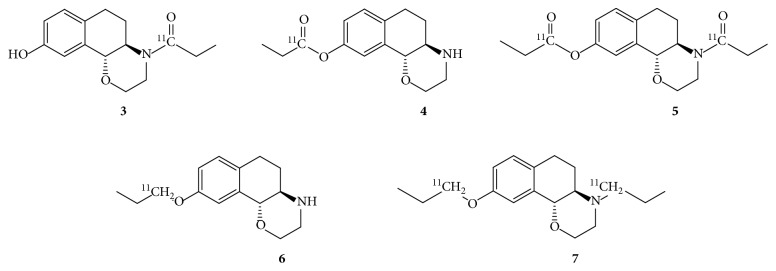
Chemical structure of the desired intermediate amide (compound **3**) as well as nonreduced form of the side products (compounds **4** and **5**) and reduced side products (compounds **6** and **7**).

Therefore, the major goal of this study was to reduce the duration of the radiosynthesis by shortening heating and cooling times and to validate the impact of these omitted temperature regulating procedures. In the end, the radiosynthetic procedure may be possible completely at room temperature. Furthermore, we aimed to evaluate each reaction step in order to understand the influence of the individual reagents on the production process. Obviously, the introduction of moisture is the most common problem, but investigating in which reaction step water was present is challenging. Therefore, the assignment of reaction intermediates (Figure 2) and their chromatographic patterns directs to a facilitated identification of the error source. Consequently, a more stable radiotracer production is obtained.

## 2. Materials and Methods

Ethylmagnesium bromide (EtMgBr, 3.0 M in diethylether, in Sure-Seal™), thionyl chloride (SOCl_2_) (99%), triethylamine (TEA; 99.5%), and tetrahydrofurane (THF, anhydrous, ≥99.9%, inhibitor-free) were purchased from Sigma-Aldrich (St. Louis, USA). Lithium aluminum hydride (LAH) (1.0 M in THF), (+)-PHNO standard, and precursor (+)-HNO hydrochloride (GMP and non-GMP) were obtained from ABX (Advanced Biochemical Compounds, Radeberg, Germany). All reagents were used without further purification. Sterile water and 0.9% saline solution were purchased from B. Braun (Melsungen, Germany). Sterile phosphate-buffered saline solution (0.021 M phosphate buffer, 0.188 M·NaCl, pH 7.7) was obtained from the Vienna General Hospital's Pharmacy (Vienna, Austria). The loop for the Grignard reactions is made of a 90 cm polyethylene (PE) tubing (fine bore polythene tubing REF 800/100/280; ID: 0.86 mm OD: 1.52 mm, Smiths Medical International Ltd.; Kent, UK). Ascarite II (20–30 mesh) was purchased from Thomas Scientific (Swedesboro, USA). Removal of acetonitrile was performed by using solid-phase extraction (SPE) cartridges (SepPak C18-plus) from Waters (Waters Cooperation; Milford, MA, USA). [^11^C]CO_2_ was produced by a GE PETtrace cyclotron 860 (General Electric Medical Systems; Uppsala, Sweden) via the ^14^N(p,*α*)^11^C nuclear reaction by irradiation of a gas target (aluminum, irradiation parameters: 16 MeV protons, 10–25 mA) filled with high-purity N_2_ (+1% O_2_) (Air Liquide Austria GmbH, Schwechat, Austria). Automated syntheses were performed on a TRACERlab™ FX C Prosynthesizer (GE Healthcare, Uppsala, Sweden) including a semipreparative HPLC system featuring a Linear Instruments Model 200 UV/Vis detector and a LaPrep HPLC pump (VWR International GmbH; Vienna, Austria). For analytical HPLC, an Agilent 1260 infinity system (Agilent Technologies GmbH; Santa Clara, USA) incorporating a quaternary pump (G1311B), a column oven (G1316A), a manual injector (G1328C), a multi wavelength UV-detector (G1365D), and a NaI(Tl)-detector from Berthold Technologies (Bad Wildbad, Germany) was used. The HPLC device is controlled by GINA Star v5.9 SP17 controlling software (Elysia-Raytest; Straubenhardt, Germany).

### 2.1. Time Optimization on the Automated Synthesizer

A reduced reaction time was achieved by omitting all heating and cooling steps with exception of CO_2_-trap heating. In particular, the target chamber was flushed twice with target gas prior to [^11^C]CO_2_ production and molecular sieves were preheated to 400°C for at least 15 min to minimize the content of nonradioactive [^nat^C]CO_2_ within the target chamber and synthesizer. Afterwards, respective lines and tubings of the synthesizer were flushed with helium. After the production of the required amount of radioactivity, [^11^C]CO_2_ was released to the synthesizer and trapped on molecular sieve. Subsequently, the trapped [^11^C]CO_2_ was released by heating to 400°C under a He stream (5 mL/min) to a PE tube, which was beforehand impregnated with a solution of ethylmagnesium bromide (EtMgBr, 1 M) in THF. The impregnation was performed by diluting 500 *μ*L EtMgBr with 1000 *μ*L THF and pushing the solution trough the loop, and then the loop was flushed with helium for around 5 sec to remove the excess of the impregnation solution. After the [^11^C]CO_2_ reaction with EtMgBr that last for around 5 min, SOCl_2_ was pushed through the tube to obtain the acid chloride and the solution was simultaneously transferred to a reactor containing a solution of (+)-HNO (1.9–2.4 mg) in TEA (50 *μ*L) and THF (400 *μ*L). The intermediate amide species was obtained after 5-6 min stirring at room temperature. Afterwards, a solution of 120 *μ*L lithium aluminum hydride (LAH) in THF (400 *μ*L) was added to reduce the intermediate amide. The reaction was quenched by addition of aqueous HCl (1 M, 900 *μ*L) and neutralized with aqueous NaOH (1 M, 900 *μ*L). The resulting suspension was filtered over cotton wool and purified by semipreparative HPLC. The collected product peak was diluted in 80 mL water and trapped on a C18-SPE cartridge. Afterwards, the product was eluted with 1.5 mL ethanol, diluted with phosphate buffered saline, and sterile filtered.

### 2.2. Evaluation of Temperatures for the Reduction Step with Lithium Aluminum Hydride (LAH)

In order to evaluate the influence of the reaction temperature on the efficacy of the reduction of the intermediate amide with lithium aluminum hydride (LAH) to (+)-[^11^C]PHNO, the synthesis was carried out using the following conditions:

−40°C (experimental set up as for −15°C)
−15°C (as previously published by our group [21])
- 22°C (shortened synthesis time and no heating and cooling)

### 2.3. Partial Runs for the Investigation of Failed Syntheses

**Figure 3:**
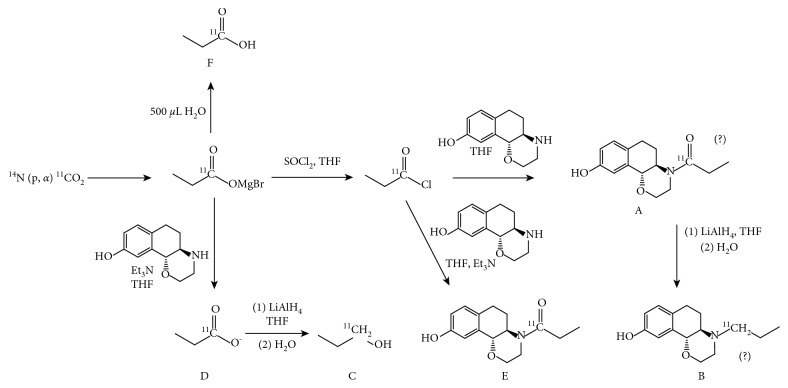
Reaction scheme of partial runs A–E for investigation of reaction intermediates formed during (+)-[^11^C]PHNO radiosynthesis.

**Figure 4:**
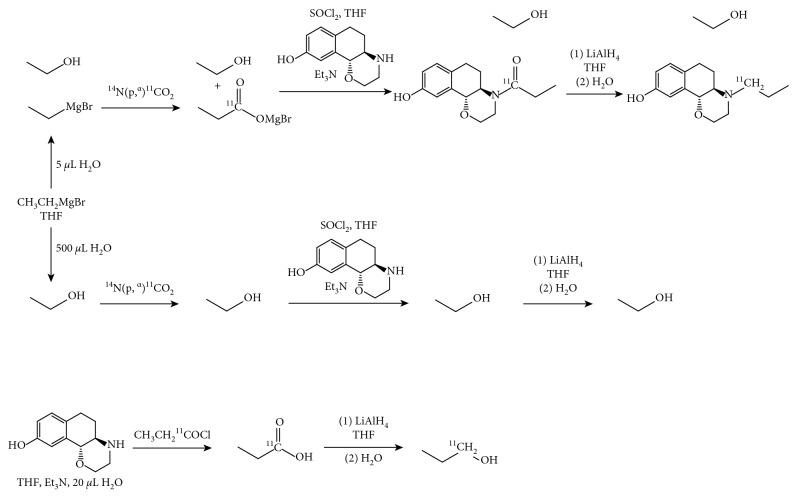
Reaction scheme of partial runs G–J for investigation of reaction intermediates formed during (+)-[^11^C]PHNO radiosynthesis.

Small-scale reactions and partial runs were conducted in borosilicate glass vials sealed with plastic septa as reaction vessel at room temperature. For all syntheses including the formation of the intermediate amide, a precursor solution of (+)-HNO (1-2 mg) in THF (400 *μ*L) and TEA (50 *μ*L) was used. The reaction was quenched by addition of H_2_O (1 mL). For the identification of byproducts and investigation of the impact of individual reagents leading to failed synthesis, partial runs were performed as depicted in Figures 3 and 4.

1. Absence of TEA: the synthesis was conducted as described above, but without the use of triethylamine. The reaction was stopped either after the amide formation (A) or at the end of the whole production scheme (B).
2. Absence of SOCl_2_: the synthesis procedure was performed either without the addition of SOCl_2_ (C) or in absence of both, SOCl_2_ and LAH (D).
3. Absence of LAH: the synthesis was stopped before LAH addition to simulate a failed reduction of the amide (E).

### 2.4. Small-Scale Reactions and Partial Runs for the Analysis of an Insufficiently Inert Atmosphere

As Grignard reactions are especially sensitive to moisture, the impact of an insufficiently inert atmosphere and therefore contamination of the reagents with traces of water was investigated.

#### 2.4.1. Influence of Moisture on the Grignard Reaction

The effect of moisture before and after trapping of [^11^C]CO_2_ in EtMgBr was investigated as follows:

- The PE tube was loaded with a mixture of 0.5 mL Grignard solution (3 M in Et_2_O) in 1 mL THF, and then the activity was released through the loop. To quench the reaction and hydrolyse the radioactive intermediate, 0.5 mL of water was pushed through the impregnated loop and the reaction mixture was analyzed (F).
- 5 *μ*L of water were added to the Grignard reagent solution and the reaction was stopped after amide formation (G) or after the reduction step (H).
- The PE tube was impregnated with the Grignard reagent solution and the loop was flushed with 0.5 mL of water. The reaction was stopped after amide formation (G1) or after the reduction step (H1).

#### 2.4.2. Influence of Moisture on Acylation

The impact of moisture on the amide formation was investigated by adding H_2_O (20 *μ*L) to a solution of (+)-HNO (1-2 mg) in THF (400 *μ*L) and TEA (50 *μ*L) prior to the addition of [^11^C]propionic acid chloride (I). Additionally, the same synthesis was performed and the resulting reaction mixture further treated with LAH (J).

### 2.5. Analytical and Semipreparative HPLC Measurements

All crude, small-scale reaction mixtures were analyzed by analytical HPLC measurements as previously published by Nics et al. [22]. The stationary phase was an X-Bridge BEH Shield RP-18, 4.6 × 50 mm, 2.5 *μ*m, 130 Å column (Waters Cooperation; Milford, MA, USA). The mobile phase consisted of solvent A (ammonia phosphate buffer (100 mM), sodium-1-octasulfonate (5 mM), pH 2.1 adjusted with H_3_PO_4_); solvent B (90% acetonitrile (ACN)/10% water); solvent C (water); and solvent D (ammonium phosphate buffer (50 mM), pH 9.3 adjusted with NaOH). The gradient started with a composition of 33% A, 17% B, 17% C, and 33% D. Subsequently, B is increased over 2 min from 17% to 34%, whereas C is reduced from 17% to 0%. The UV/Vis signal was detected at a wavelength of 280 nm with a reference wavelength of 450 nm. The initial flow rate (1.5 mL/min) was decreased after 25 s to 1.0 mL/min. The retention time of the radioactive peaks was compared to the reference standard of (+)-PHNO in order to identify the respective product peak.

A part of the partial runs was additionally investigated on a semipreparative HPLC system with a Phenomenex Luna C18 column (250 × 10 mm, 10 *μ*m; Phenomenex Ltd., Aschaffenburg, Germany) as a stationary phase. The mobile phase consisted of 25 mM phosphate-buffered saline (PBS) (pH 7.0)/ACN (60/40 v/v) with a flow rate of 5.8 mL/min. The UV/V is signal was measured at 254 nm. The peaks were collected and correlated with the respective peaks of the analytical HPLC.

### 2.6. Assignment of the Chromatographic Peaks of [^11^C]CO_2_ and [^nat^C]Propionic Acid

[^11^C]CO_2_ was trapped in THF, and the solution was analyzed by analytical HPLC. The intermediate product propionic acid was injected to the analytical HPLC, and its retention time was compared to the radioactive impurity peaks of the crude mixture.

### 2.7. Synthesis of Compound **3** Using [^nat^C]CO_2_

The intermediate amide **3** was synthesized by using [^nat^C]CO_2_ gas that was bubbled through an EtMgBr solution (15 *μ*L, 3.0 M in Et_2_O) in THF (200 *μ*L) for 3 min under He atmosphere. Afterwards, a solution of SOCl_2_ (5 *μ*L) in THF (400 *μ*L) was added to generate the acyl chloride. After 4 min, the precursor solution of (+)-HNO (5 mg, non-GMP) in TEA (50 *μ*L) and THF (200 *μ*L) was added to the reaction mixture. The resulting suspension was extracted three times with CH_2_Cl_2_ (about 500 *μ*L). The combined organic phases were injected into the semipreparative HPLC system. The collected peaks were analyzed by analytical HPLC and HRMS (ESI-MS: HNO (C_12_H_16_NO_2_ [M + H^+^] calcd. 206.118, found 206.118), **3** (or **4**) (C_15_H_20_NO_3_ [M + H^+^] calcd. 262.144, found 262.143, C_15_H_19_NO_3_Na [M + Na^+^] calcd. 284.126, found 284.125), and **5** (C_18_H_24_NO_4_ [M + H^+^] calcd. 318.171, found 318.170, C_18_H_23_NO_4_Na [M + Na^+^] calcd. 340.153, found 340.152).

### 2.8. Characterization of Intermediate Compounds and Side Products

Characterization and identification of the compounds separated by HPLC was performed by high-resolution mass spectrometry (HRMS) on a Bruker maXis UHR-TOF device (electrospray ionization (ESI); qQ-TOF, mass accuracy < 5 ppm) in either positive or negative mode depending on the respective molecular structure.

### 2.9. Definition of Yield Determination

According to the consensus of nomenclature, the activity yield is defined as the amount of product that is obtained after a synthesis in MBq or GBq and is not corrected for decay. This term is related to the starting activity of [^11^C]CO_2_ and can be expressed in [%] as radiochemical yield not corrected for decay [23].

### 2.10. Statistical Analysis

Study data were obtained from all syntheses performed in our department between 01/2017 and 08/2017 with starting activity of 100–150 GBq at the end of bombardment (EOB) of [^11^C]CO_2_. Values are given as mean values ± SD as calculated with Microsoft Excel 2010 if not stated otherwise. Statistical analysis was performed by using a *t* test as implemented in Graph Pad Prism 6 (GraphPad Software, La Jolla, USA).

## 3. Results and Discussion

### 3.1. Reduction of the Radiosynthesis Duration for an Improved Activity Yield

**Figure 5:**
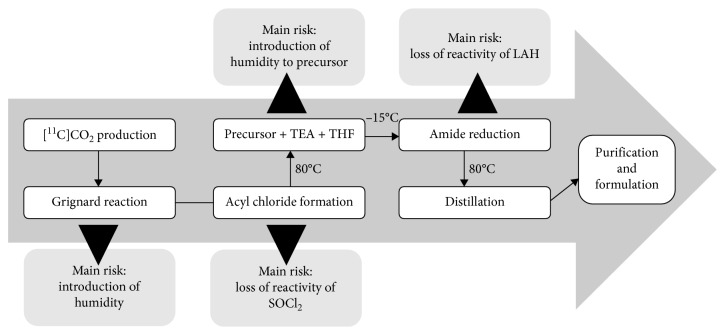
Reaction steps of (+)-[^11^C]PHNO production according to Rami-Mark et al. [21] with the corresponding main risk for each reaction step.

The previously described radiosynthetic process for (+)-[^11^C]PHNO preparation by Rami-Mark et al. involves a heating step to 80°C for the acylation reaction, followed by the addition of LAH at −15°C [21]. Afterwards, the residual THF is removed via distillation (Figure 5).

**Figure 6:**
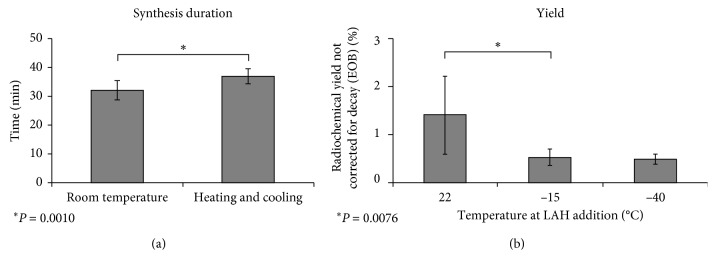
(a) Comparison of the synthesis duration of (+)-[^11^C]PHNO synthesis at room temperature (*n* = 16) and according to our previously published method including heating and cooling (*n* = 9). (b) Influence of the temperature at LAH addition on the isolated radiochemical yield not corrected for decay (22°C: *n* = 16, −15°C: *n* = 9, −40°C: *n* = 3).

In the improved method, all reaction steps of (+)-[^11^C]PHNO synthesis were performed at room temperature. In detail, the acylation with [^11^C]propionic acid chloride was performed without heating and cooling as well as the addition of LAH. In this study, the improved synthesis (*n* = 16) was compared with two experimental setups: (1) (+)-[^11^C]PHNO synthesis was conducted as previously described by our group (*n* = 9) [19]. (2) LAH was added at −40°C as described by Wilson et al. (*n* = 3) [12]. Performing all reaction steps at room temperature realized an average reduction of the overall synthesis time by around 5 min (approximately 13% in comparison to the previously published synthesis [21]) as described in Figure 6. This time reduction is especially beneficial for the production of a ^11^C-radiotracer as it impedes loss of radiolabeled product by decay of around 15%. As a result, the activity yield was increased in comparison to previously published synthetic procedures. Synthesis at room temperature led to a significantly increased isolated radiochemical yield not corrected for decay of 1.4 ± 0.8%. All yields within this manuscript were calculated by referring the product activity to the activity after the end of bombardment. [23] Indeed, the experiments in which the reduction was carried out at −15 or −40°C resulted in 0.53 ± 0.17% and 0.50 ± 0.11% isolated radiochemical yield without decay correction, respectively (Figure 6). Thus, this study clearly showed that (+)-[^11^C]PHNO synthesis can be successfully performed at room temperature without a forfeit of activity yield. The starting activities and activity yields of the experiments are given in Table 1.

The success rate of the synthesis at room temperature exceeds the success rate of our previously published method by Rami-Mark et al. (Table 1) [21]. The success rate of LAH addition at −40°C is 100%, but it should be considered that the number of performed experiments is only 3.

**Figure 7:**
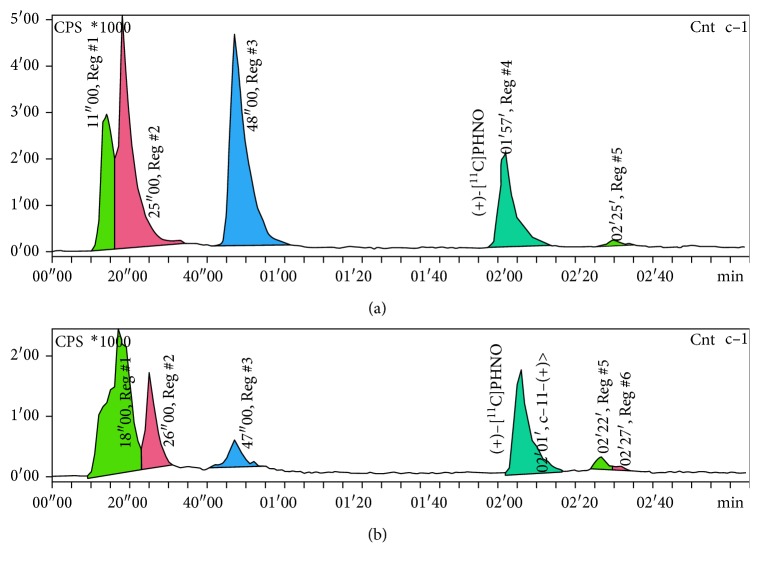
Analytical HPLC chromatograms of the crude reaction mixtures obtained by addition of LAH at room temperature (a) or at −40°C (b).

The limiting parameter of the reaction at room temperature is the reactor volume as the solvent is not evaporated and an overflow of the reaction mixture must be avoided. If an adequate reactor is available, this facilitated four-step synthesis procedure can be performed in every radiochemistry laboratory that is equipped with a cyclotron. All experiments showed a similar pattern of the crude reaction mixture in semipreparative as well as analytical HPLC chromatograms but a different intensity of the peaks (Figure 7). The peaks with a retention time of 0–26 s are hydrophilic compounds like [^11^C]CO_2_ and [^11^C]propionic acid. The species at 47 s and 48 s are probably the carbonyl intermediates. The peak at a retention time range of 1 min 55 sec to 2 min 10 sec originate from (+)-[^11^C]PHNO. However, after purification by semipreparative HPLC, (+)-[^11^C]PHNO could be obtained in an excellent purity of >95% for all applied methods.

### 3.2. Partial Runs for the Investigation of Failed Synthesis

**Figure 8:**
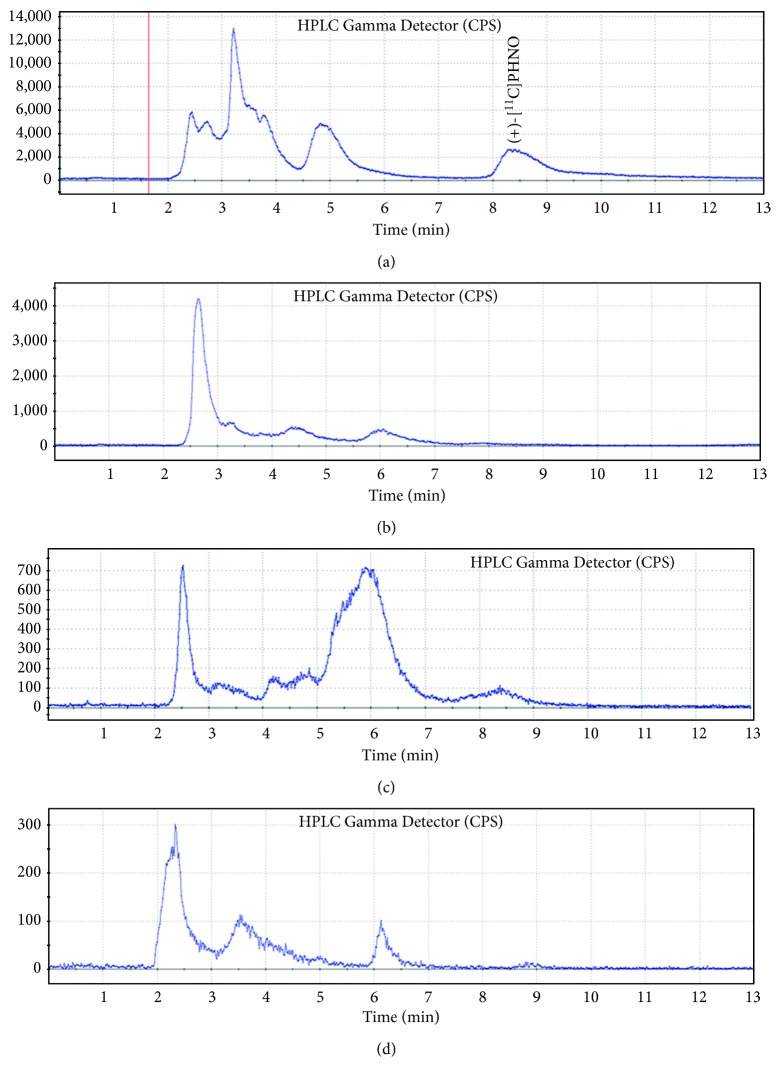
Chromatogram of semipreparative HPLC of full synthesis at room temperature (a), route A synthesis without TEA and LAH (b), route E without LAH (c), and route F in which the reaction is quenched after Grignard reaction (d).

Radioactive reaction intermediates and byproducts were found at retention times of 2-3 min, 3-4 min, 4.8 min, 6 min, and 8 min within semipreparative HPLC, as well as of 20 s, 45 s, and 2.5 min in analytical HPLC, respectively, for successful (+)-[^11^C]PHNO synthesis (Figures 7(a) and 8(a)). Partial runs of the synthesis were performed to identify the byproducts and therefore the respective error sources.

#### 3.2.1. Absence of Triethylamine

The syntheses were performed in absence of triethylamine, and therefore, the precursor (+)-HNO was still protonated. Syntheses were either stopped at the intermediate amide (route A) or conducted until the end of synthesis (route B).

In route A, semipreparative HPLC showed the main species at a retention time of 2.5–3 min (Figure 8(b)), and in analytical HPLC, the main peak was observed at short retention times (15–20 s and comprised about 66.8 ± 0.2%), usually representing small hydrophilic compounds, like [^11^C]CO_2_ and [^11^C]propionic acid. Further peaks with smaller intensities were observed at longer retention times (4.5 min and 6 min in semipreparative HPLC) showing minor amounts of potential carbonyl byproducts (compound **3** or **4**) resulting from an insufficient deprotonation of the precursor, which impedes the acylation process.

Reaction scheme B, which included the reduction step with LAH, shows similar chromatographic pattern. However, no product formation was observed showing that deprotonation of (+)-HNO is pivotal for a successful (+)-[^11^C]PHNO synthesis. In conclusion, a chromatogram with intensive signals at early retention times, poor signal at retention times of 5–6.5 min, and no formation of product may originate from nonintact Et_3_N.

#### 3.2.2. Absence of SOCl_2_

All experiments, which were performed without SOCl_2_, were either stopped after theoretical amide formation (C) or after the reduction step (D). Semipreparative HPLC showed only peaks with retention time <4 min and no product formation. A similar picture was observed for analytical HPLC. Thus, complete absence of high retention-time peaks indicates inactivated or decomposed SOCl_2_.

#### 3.2.3. Absence of LAH

Simulation of a failed LAH-reduction step was done by stopping prior to the reduction step (E). Here, a radioactive peak at a retention time of 6 min could be observed in semipreparative HPLC. This peak also occurred in other experiments without LAH (route B) and therefore potentially represents a carbonyl species, namely, **3**, **4,** or **5**. Furthermore, the signal could be assigned to the 45 s peak in analytical HPLC being the most prominant one with an intensity of 51 ± 22%. A highly dominant peak in the semipreparative HPLC chromatogram at 6 min without product formation concludingly points to a failed LAH-reduction. As a consequence, a new bottle of LAH should be used for following syntheses and special attention should be paid to the inert atmosphere.

### 3.3. Small-Scale Reactions and Partial Runs for the Analysis of an Completely Inert Atmosphere

For investigating the influence of minor amounts of moisture on the Grignard reagent, following experiments were performed:

- Hydrolysis of the Grignard reagent after the addition of [^11^C]CO_2_ (route F)
- Adding water to the Grignard reagent before addition of [^11^C]CO_2_ (routes G and H)

In addition, the moisture sensitivity of the acylation was tested by intentionally adding water to the precursor solution (routes I and J).

#### 3.3.1. Influence of Moisture on the Grignard Reaction

The route F led to semipreparative chromatographic peaks at retention times of 2-3 min, 3–5 min, and 6.0–6.5 min. In this respect, analytical HPLC displayed a main signal at a retention time of 15–20 s (75 ± 7%). Surprisingly, we also observed peaks at 45 s and 2.5 min, although the expected species, propionic acid and [^11^C]CO_2_, are supposed to elute at earlier retention times.

Simulation of a Grignard reaction under an insufficiently inert atmosphere on the acylation was performed by directly adding 5 *μ*L water to the Grignard reagent (G and G1), whereas for a complete quenching of the reaction, 500 *μ*L water was added (H and H1).

When the reaction process was stopped after amide formation, an intensive peak at 6 min was visible in semipreparative HPLC for an addition of 5 *μ*L, whereas this peak was not detected for 500 *μ*L. Hence, the occurrence of this peak shows that 5 *μ*L water is not sufficient for quenching the Grignard reaction completely due to stoichiometric reasons: 15 mmol of EtMgBr are used for the reaction and 0.28 mmol H_2_O was added. Therefore, as a relatively huge amount of water is needed for a fail in synthesis, moisture alone will not lead to a quenched reaction. This effect could be confirmed by conducting the complete reaction process. Addition of 5 *μ*L water to the Grignard solution (route H) leads to minor amounts of (+)-[^11^C]PHNO, whereas the addition of 500 *μ*L of water (H1) completely quenched all further reactions. As a consequence, the presence of peaks with a retention time less than 4 min in semipreparative HPLC may originate from a decomposed Grignard reagent.

**Figure 9:**
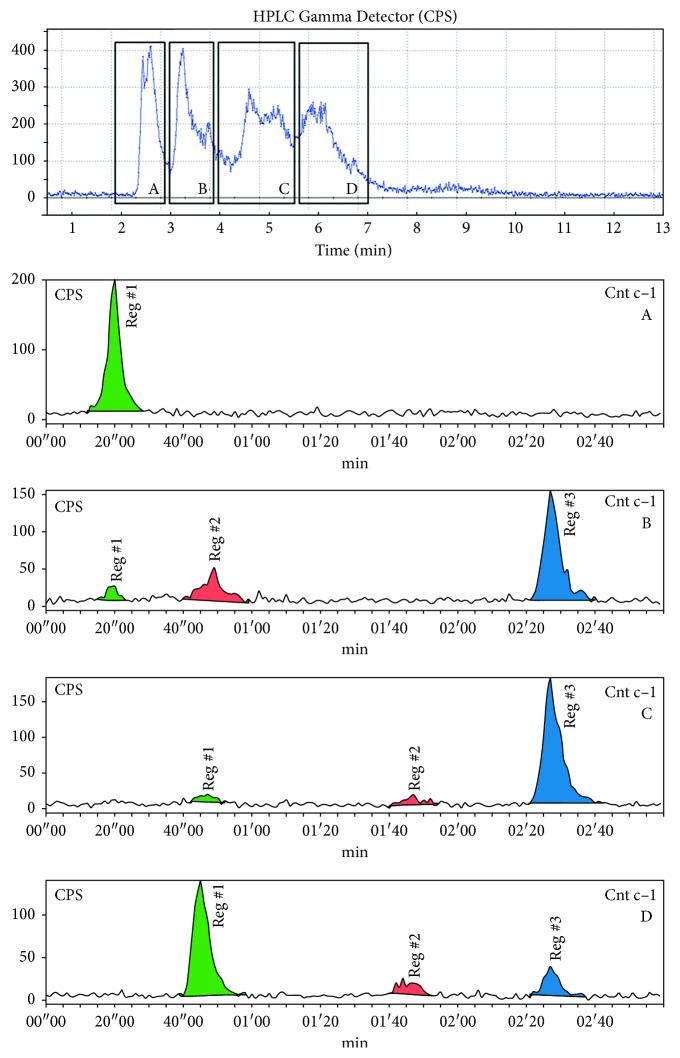
Semipreparative HPLC chromatogram of partial run G and the corresponding analytical chromatograms of the respective peak.

Besides, the peaks of the semipreparative HPLC and analytical HPLC were correlated to each other as follows (Figure 9): retention times below 3 min (semipreparative HPLC) corresponds to a peak at 20 s (analytical HPLC), the peaks between 3 min and 5 min 30 s are similar to the one at 2 min 30 s, and the 6 min peak to the one at 45 s. Accordingly, the two peaks of 3–5 min 30 s and 6 min in semipreparative HPLC switched their retention order in the analytical run.

#### 3.3.2. Influence of Moisture on Acylation

Here, 20 *μ*L water was added to the precursor solution (400 *μ*L THF, 50 *μ*L TEA, 0.9–1.4 mg (+)-HNO)) prior to addition of intact propionic acid chloride. The synthesis was stopped after formation of the amide (I) or after the reduction (J). For partial run I, the ratio of the reaction intermediates was as follows: 15–20 s and 45 s with 37.5% and 34.0%, respectively. Due to the high intensity of the peak at 45 s, it can be assumed that the reaction was not completely quenched and one of the carbonyl species was partly formed. Likewise, semipreparative HPLC revealed signals at 4–7 min indicating carbonyl formation. Performing the entire synthetic route for (+)-[^11^C]PHNO production (J) leads to a peak at a retention time similar to (+)-[^11^C]PHNO (9 min) with low intensity. However, analytical HPLC showed a peak at 1 min 26 s with 8.3% conversion but no signal at 2 min for the product. This chromatographic pattern is quite similar to the one observed for the partial run without TEA.

### 3.4. Assignment of the Chromatographic Peaks of [^nat^C]Propionic Acid and [^11^C]CO_2_

**Figure 10:**
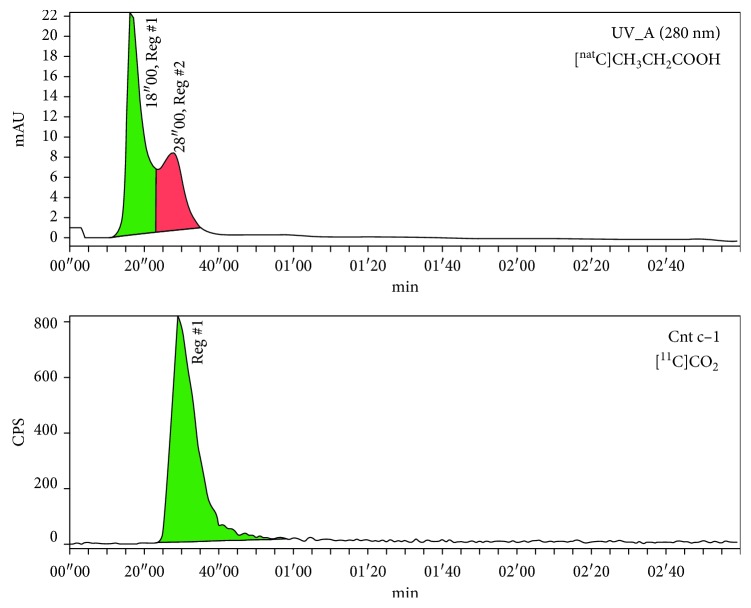
(a) UV/Vis chromatogram of [^nat^C]propionic acid. (b) Radio-channel chromatogram of [^11^C]CO_2_.

One intermediate of (+)-[^11^C]PHNO synthesis is [^11^C]propionic acid, which was identified by comparison of the nonradioactive reference compound [^nat^C]propionic acid. The analytical chromatogram showed a signal at 15–40 s at 280 nm wavelength (Figure 10). Moreover, [^11^C]CO_2_ was trapped in THF to assign the respective peak as well. The signal was found at a retention time of 20–40 s similar to [^nat^C]propionic acid (Figure 10). Hence, both compounds, [^11^C]CO_2_ as well as [^11^C]propionic acid, are not base line separated and were present in nearly every analytical chromatogram. Those results support the previously obtained data, showing that the exclusive presence of early eluting peaks indicates problems within the first reaction steps preventing amide formation.

### 3.5. Synthesis of Compound **3** Using [^nat^C]CO_2_ for Assignment of HPLC Peaks

The cold synthesis of the intermediate amide resulted in a crude reaction mixture that was transferred onto semipreparative HPLC. The fractions were collected and measured by HRMS (high-resolution mass spectrometry). The respective signal of the precursor, as well as of compounds **3** or **4** and **5** could be identified (see Materials). As **3** and **4** have the same molecular weight, those two molecules cannot be differentiated by mass spectrometry. Besides, the formation of byproducts **5** and **7** during radiosynthesis is very unlikely due to substoichiometric ratio in radiosynthesis.

### 3.6. Establishment of a Troubleshooting Protocol

**Figure 11:**
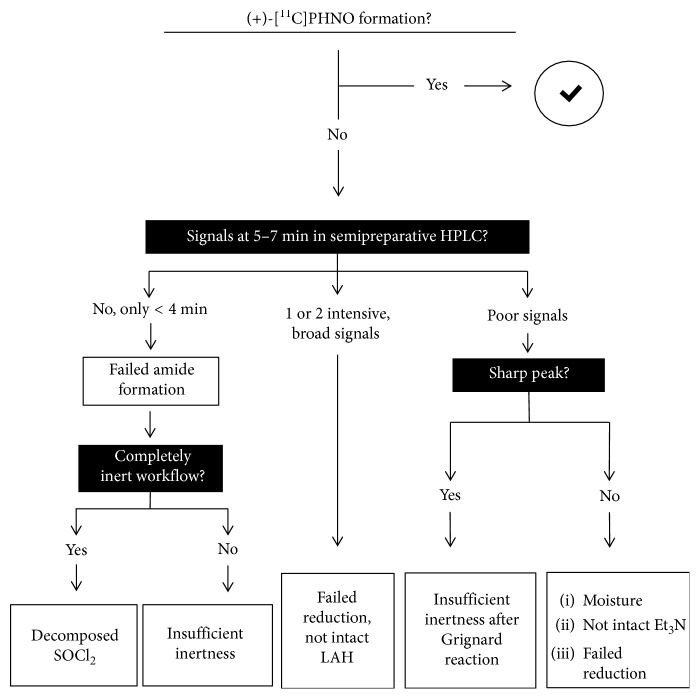
Schematic decision tree aiding troubleshooting after a failed (+)-[^11^C]PHNO formation.

As shown in this study, failure of (+)-[^11^C]PHNO radiosynthesis can be attributed to various reasons, but the chromatogram of the semipreparative HPLC provides information on error sources. Based on our results, we created a decision scheme that helps troubleshooting in case of a failed (+)-[^11^C]PHNO production (Figure 11). The presence of intensive and broad peaks in the region of 5–7 min shows a successful carbonylation but a failed reduction to (+)-[^11^C]PHNO. Consequently, the LAH should be renewed. If there is at least one broad peak with low intensity in this range, the amide formation was incomplete, which is mainly attributed to moisture. In addition, a single, sharp peak between 5 and 7 min suggests the presence of water after the Grignard reaction. A chromatogram that is missing those signals indicates a failure in the coupling and can be assigned to a poor inert atmosphere or decomposed SOCl_2_. Hence, the SOCl_2_ should be changed. This scheme will facilitate troubleshooting and lead to an improved rate of successful (+)-[^11^C]PHNO.

## 4. Conclusion

The complex synthesis of (+)-[^11^C]PHNO is a challenge for every radiochemist in terms of time efficiency, reduction of failed syntheses, and error evaluation. Here, we present a new method allowing a tremendous reduction of the radiosynthetic duration of approximately 5 min by omitting heating and cooling steps, which enhances the activity yield significantly. Moreover, the investigation of side products and intermediate species facilitates the error evaluation after a failed synthesis. Accordingly, we propose a decision tree to support troubleshooting and facilitating a stable and continuous radiotracer production that is a necessity for clinical studies.

## Figures and Tables

**Table 1:** Starting activity, activity yield, number of experiments, and success rate of the studied experimental setups.

Temperature at LAH addition (°C)	Starting activity (EOB) (GBq)	Activity yield (GBq)	*n*	Success rate (%)
22	120 ± 15	1.7 ± 1.0	16	89
−15	122 ± 13	0.7 ± 0.2	9	82
−40	113 ± 8	0.6 ± 0.1	3	100

## Data Availability

The synthesis and the preparative HPLC data used to support the findings of this study are available from the corresponding author upon request. The quality control data used to support the findings of this study are included within the article.
